# Transcription factor CREB3L1 mediates cAMP and glucocorticoid regulation of arginine vasopressin gene transcription in the rat hypothalamus

**DOI:** 10.1186/s13041-015-0159-1

**Published:** 2015-10-26

**Authors:** Mingkwan Greenwood, Michael P. Greenwood, Andre S. Mecawi, Su Yi Loh, José Antunes Rodrigues, Julian F. R. Paton, David Murphy

**Affiliations:** School of Clinical Sciences, University of Bristol, Dorothy Hodgkin Building, Whitson Street, Bristol, BS1 3NY England; School of Medicine of Ribeirão Preto, University of São Paulo, Ribeirão Preto, Brazil; Department of Physiology, University of Malaya, Kuala Lumpur, 50603 Malaysia; Department of Physiological Sciences, Biology Institute, Federal Rural University of Rio de Janeiro, Seropedica, Rio de Janeiro Brazil; School of Physiology and Pharmacology, University of Bristol, Bristol, BS8 1TD England

**Keywords:** Transcription, Forskolin, Dexamethasone, Hyperosmotic, Magnocellular neuron, Supraoptic nucleus, Paraventricular nucleus, AtT20, Rat, Organotypic culture

## Abstract

**Background:**

Arginine vasopressin (AVP), a neuropeptide hormone that functions in the regulation of water homeostasis by controlling water re-absorption at kidneys, is synthesised in supraoptic nucleus and paraventricular nucleus of the hypothalamus. An increase in plasma osmolality stimulates secretion of AVP to blood circulation and induces AVP synthesis in these nuclei. Although studies on mechanism of AVP transcriptional regulation in hypothalamus proposed that cAMP and glucocorticoids positively and negatively regulate *Avp* expression, respectively, the molecular mechanisms have remained elusive. Recently, we identified CREB3L1 (cAMP-responsive element binding protein 3 like 1) as a putative transcription factor of *Avp* transcription in the rat hypothalamus. However the mechanism of how CREB3L1 is regulated in response of hyperosmotic stress in the neurons of hypothalamus has never been reported. This study aims to investigate effect of previously reported regulators (cAMP and glucocorticoid) of *Avp* transcription on transcription factor CREB3L1 in order to establish a molecular explanation for cAMP and glucocorticoids effect on AVP expression.

**Results:**

The effect of cAMP and glucocorticoid treatment on *Creb3l1* was investigated in both AtT20 cells and hypothalamic organotypic cultures. The expression of *Creb3l1* was increased in both mRNA and protein level by treatment with forskolin, which raises intracellular cAMP levels. Activation of cAMP by forskolin also increased *Avp* promoter activity in AtT20 cells and this effect was blunted by shRNA mediated silencing of *Creb3l1*. The forskolin induced increase in *Creb3l1* expression was diminished by combined treatment with dexamethasone, and, in vivo, intraperitoneal dexamethasone injection blunted the increase *in Creb3l1* and *Avp* expression induced by hyperosmotic stress.

**Conclusion:**

Here we shows that cAMP and glucocorticoid positively and negatively regulate *Creb3l1* expression in the rat hypothalamus, respectively, and regulation of cAMP on AVP expression is mediated through CREB3L1. This data provides the connection between CREB3L1, a newly identified transcription factor of AVP expression, with the previously proposed mechanism of *Avp* transcription which extends our understanding in transcription regulation of *Avp* in the hypothalamus.

## Background

Expression of the gene encoding the neuropeptide hormone arginine vasopressin (AVP), which functions in water homeostasis, is found in the magnocellular neurons (MCNs) of supraoptic nucleus (SON) and paraventricular nucleus (PVN). The AVP prohormone is synthesised in the cell body and subsequently transported to the axon terminal in the posterior pituitary where it is stored [1]. Secretion is activated by hyperosmotic conditions through excitatory inputs from circumventricular organs [2, 3]. An increase of plasma osmolality activates secretion of AVP into the blood circulation to control water re-absorption at the kidney [4].

Whilst the regulation of *Avp* gene expression in the hypothalamus has been studied for over three decades, ever since the cloning of the gene itself [5], the molecular details of the signaling and transcriptional mechanisms involved have remained elusive. That said, some evidence suggests that the cAMP/PKA pathway positively regulates expression of *Avp*, possibly through the phosphorylated cAMP responsive element-binding protein (CREB) [6, 7]. An increase in cAMP and phosphorylated CREB was observed in the SON during osmotic stimulation [7, 8]. Activation of cAMP by forskolin (FSK; an adenylate cyclase activator) in dissociated hypothalamic primary culture, and treatment of fetal hypothalamic cultures with a protein kinase A activator, served to increase *Avp* expression [6, 9]. Moreover the *in vitro* analysis of the rat *Avp* promoter in the human choriocarcinoma cell line JEG-3 identified two cAMP responsive element (CRE)-sites (−227/-220 and −123/-116), and the expression of a dominant negative form of CREB decreased *Avp* promoter activity, suggesting that the CREB protein regulates *Avp* transcription [10]. However, the function of CREB as a transcription factor for *Avp* was not confirmed *in vivo* [11]. In addition, a role for the immediate early gene Fos/Jun transcription factor family in *Avp* transcription has also been proposed through binding to an activator protein 1 (AP1) regulatory sequence in the *Avp* promoter [12–14].

On the other hand, glucocorticoids, the end product of hypothalamo-pituitary-adrenal axis, were shown to negatively regulate the expression of *Avp* in the hypothalamus as part of a negative-feedback loop. Withdrawal of glucocorticoids by adrenalectomy results in increased *Avp* mRNA expression in the PVN [15, 16], which can be reversed by glucocorticoid replacement [17]. There is also evidence supporting a role for glucocorticoids in the control of water homeostasis. In 2004, Durlo and colleagues reported increase of plasma corticosterone concentration, an event well known to occur in stress conditions, in response to hypervolemia and hyperosmolarity [18]. Moreover long-term hypoosmolality has been shown to increase glucocorticoid receptor expression in vasopressinergic MCNs of the SON, suggesting that glucocorticoids can inhibit AVP synthesis in response to osmotic cues [19]. *In vitro* promoter analysis studies have reported negative effects of glucocorticoid on *Avp* promoter activity [10], but the regulatory mechanisms have been unclear.

Recently, we identified the basic leucine zipper transcription factor CREB3L1 (cAMP-responsive element binding protein 3 like 1; also called OASIS) as a putative transcriptional regulator of *Avp* expression in the rat hypothalamus [20]. CREB3L1, an endoplasmic reticulum (ER) and Golgi-resident protein, is a transcription factor in CREB/ATF family. Its structure is similar to ER stress inducer ATF6 (Activating transcription factor 6) which contains N-terminal activation, transmembrane and C-terminal domains. CREB3L1 is activated by regulated intramembrane proteolysis, resulting in the release of an N-terminal activating domain that is transported to the nucleus, where it regulates the transcription of target genes [21]. Activation of CREB3L1 was shown to be induced by ER stress in many physiological models but the mechanism of activation in neurons of the hypothalamus has never been reported.

Interestingly, cAMP was recently shown to activate expression of *Creb3l1* in trophoblast cell line BeWo [22]. Thus in this study, we tested the hypothesis that expression of CREB3L1, a newly identified transcription factor of AVP may be regulated through cAMP pathway and glucocorticoid, the previously proposed regulators of AVP expression, and the effects of these regulators on AVP transcription may be mediated through CREB3L1 expression.

## Results

### Effect of cAMP and glucocorticoid on *Creb3l1* expression

**Fig. 1:**
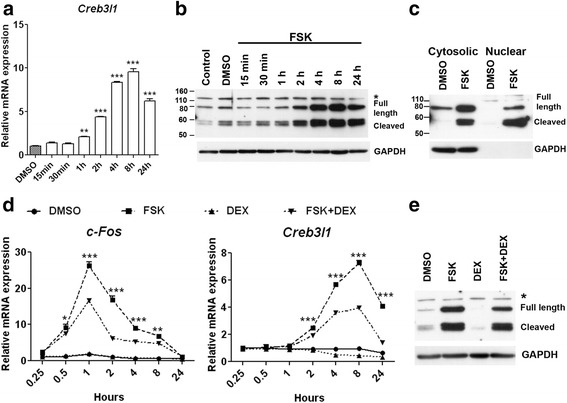
cAMP and glucocorticoid regulate *Creb3l1* expression. AtT20 cells were used to study regulation of *Creb3l1*. **a**–**c** AtT20 cells were treated with 10 μM FSK or vehicle (DMSO). **a** *Creb3l1* mRNA expression was observed by qPCR at various time points (*n* = 3; One way ANOVA). **b**–**c** Immunoblotting was performed to examine protein expression level of CREB3L1 in both **b** total protein extracts and **c** cytosolic/nuclear extracts. **d**–**e** AtT20 cells were treated with vehicle (DMSO), 10 μM FSK and/or 100nM DEX at various time points. **d** Effect of glucocorticoid on *c-Fos* and *Creb3l1* expression was examined by qPCR (*n* = 3; Two way ANOVA). **e** Immunoblotting of CREB3L1 was performed at 24 h after treatment. GAPDH was used as an internal control for immunoblotting. * in B and E indicates non-specific band in immunoblotting. * in D indicate significant from FSK + DEX compared to FSK group. Scale bar, 20 μm; Error bar, +SEM; *,*p *< 0.05, **, *p* < 0.01; ***, *p* < 0.001; FSK, forskolin; DEX, dexamethasone

To investigate molecular details of the cAMP and glucocorticoid effects on *Creb3l1* expression, we used the mouse pituitary AtT20 cell line as a model. The expression of *Creb3l1* mRNA in AtT20 cells was investigated by qPCR. Treatment with cAMP activator (10 μM forskolin; FSK) increased *Creb3l1* mRNA expression in a time-dependent manner (Fig. 1a). This increase was maintained, although at a reduced level, at the 24 h time point. Immunoblotting also revealed an increase in CREB3L1 protein abundance following FSK treatment (Fig. 1b). Both the cleaved form and full length of CREB3L1 are clearly observed 2 h after FSK treatment, and levels of both increased in a time-dependent manner, in parallel with mRNA abundance. We also performed immunoblotting on fractionated cytosolic and nuclear extracts of AtT20 cells treated with FSK for 24 h (Fig. 1c). An increase of both forms of CREB3L1 was observed in the cytosolic portion of FSK-treated cells, with the cleaved form being majorly increased in the nuclear fraction. This suggests that the amount of CREB3L1 is increased following FSK treatment, and that the full-length protein is subsequently processed, with the N-terminal active cleavage product being transported to the cell nucleus.

Next, the effect of glucocorticoid was investigated in AtT20 cells using a potent glucocorticoid medication, dexamethasone (DEX). We first characterized the effect of FSK and/or DEX on AtT20 cells using the expression of *c-Fos*, an immediate early gene that requires cAMP for activation [23], as a marker for cAMP activation (Fig. 1d). FSK treatment rapidly, but transiently, increased *c-Fos* mRNA levels. The maximal induction was reached at 1 h, with levels returning to baseline by 24 h. DEX treatment had no effect on basal *c-Fos* mRNA level but reduced the effect of FSK induction. DEX effects on *Creb3l1* expression were investigated by qPCR (Fig. 1d). Treatment with DEX progressively decreased *Creb3l1* mRNA expression under basal conditions. As before (Fig. 1a), FSK treatment increased *Creb3l1* transcript abundance, while DEX attenuated this effect of FSK on *Creb3l1* mRNA expression. CREB3L1 immunoblotting showed an increase of CREB3L1 following 24 h of FSK treatment. Lower levels of CREB3L1 were observed following DEX treatment compared to controls, and the combination of FSK and DEX reduced CREB3L1 protein levels compared to FSK treatment alone (Fig. 1e).

### Effect of FSK and DEX treatment on *Creb3l1* in organotypic cultures of rat hypothalamus

**Fig. 2:**
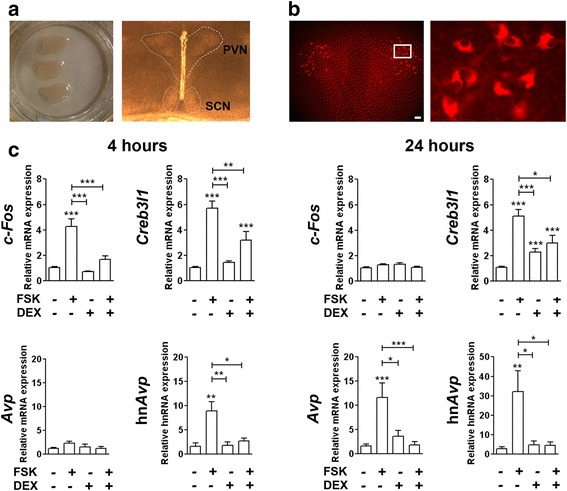
Effect of cAMP activation and glucocorticoid in organotypic culture of rat hypothalamus. **a** Rat hypothalamic explants were cultured on Millicell membranes. An observation under bright field microscope showed the area of PVN and SCN are intact. **b** Immunofluorescent staining of AVP-NPII was performed at day 14 to demonstrate survival of vasopressinergic neurons in the culture. The high magnification image of the area in the box is shown. **c** The mRNA expression of hypothalamic explants in response to treatment of 10 μM FSK and/or 100nM DEX for 4 h (*n* = 5-7) and 24 h (*n* = 8-15) was examined by qPCR. Scale bar, 100 μm; Error bar + SEM; *, *p* < 0.05; **, *p* < 0.01; ***, *p* < 0.001 (One way ANOVA); FSK, forskolin; DEX, dexamethasone; PVN, paraventricular nucleus; SCN, suprachiasmatic nucleus. hn*Avp*, arginine vasopressin heteronuclear RNA

To investigate the effect of cAMP and glucocorticoid on *Creb3l1* expression in the neurons of hypothalamus, we investigated the effect of FSK and DEX in the organotypic culture extracted from rat hypothalamus (Fig. 2a). This model was successfully used to study activation of *Avp* by cAMP [24]. Immunofluorescent staining using an antibody recognizing AVP NP-II confirmed the survival of AVP neurons in these cultures (Fig 2b). The effects of FSK and DEX treatments on *Creb3l1* expression in the organotypic cultures were investigated by qPCR. Cultures were treated with FSK and/or DEX for 4 and 24 h (Fig. 2c). An increase in *c-Fos* expression was observed at the early time point of treatment (4 h), but not 24 h, after FSK treatment. DEX had no significant effect on basal level of *c-Fos* mRNA at both time points; however it inhibited the effect of FSK-induced *c-Fos* expression at the 4 h time point. *Creb3l1* expression increased following FSK treatment at both 4 h and 24 h time points. Interestingly, DEX treatment alone increased *Creb3l1* expression at 24 h, not the 4 h time point, but inhibited FSK-mediated induction. As expected, FSK treatment increased hn*Avp* levels, an effect also inhibited by DEX. *Avp* mRNA only increased at the 24 h FSK treatment, where DEX also inhibited the effect of FSK.

### Activation of cAMP pathways modulates *Avp* transcription through CREB3L1

**Fig. 3:**
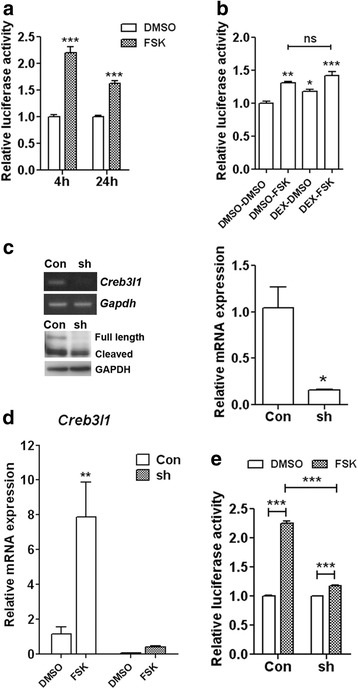
cAMP-activated AVP promoter activity is modulated through *Creb3l1.* **a**-**b** AtT20 cells were transfected with 1 kb rat *Avp* promoter construct. At 24 h after transfection the cells were **a** treated with 10 μM FSK for 4 and 24 h or **b** pre-treated with 100nM DEX or DMSO for 2 h and followed by 4 h treatment of 10 μM FSK or DMSO and luciferase activity was measured (*n* = 3). **c**–**e** The *Creb3l1*-knockdown AtT20 cell line was produced in parallel with a control non-targeting shRNA cell line by transduction of lentivirus containing *Creb3l1* or control non-targeting shRNA and further selected with puromycin. **c** The level of *Creb3l1* knockdown was investigated by qPCR and immunoblotting in the AtT20 shRNA cell lines (*n* = 3). **d** The effect of FSK on *Creb3l1* mRNA expression was examined by qPCR in control non-targeting and *Creb3l1* knockdown cell lines (*n* = 3). **e** Luciferase assays were performed in control and *Creb3l1*-knockdown cell lines using 1 kb *Avp* promoter construct driving luciferase reporter gene (*n* = 3-4). Error bar + SEM; *, *p* < 0.05; **, *p* < 0.01; ***, *p* < 0.001 (Two way ANOVA for A, D and E, One way ANOVA for B and independent sample unpaired Student’s *t*-tests for C). FSK, forskolin; Con, Control; sh, shRNA; h, hour; ns, no significant difference

We asked if cAMP pathways could activate the *Avp* promoter in AtT20 cells. AtT20 cells were transfected with luciferase reporter gene plasmids containing 1 kb of the rat *Avp* promoter, and then treated with FSK for 4 h and 24 h (Fig. 3a). Robust increases in luciferase activity were seen in FSK treated cells compared to vehicle controls at both time points with a slight decrease of activity at 24 h compared to 4 h. Effect of DEX on AVP promoter activity was also investigated (Fig. 3b). Surprisingly we observed an increase of luciferase activity in response to DEX treatment and a combination of FSK and DEX treatment.

We then tested the hypothesis that the cAMP induced up-regulation of *Avp* promoter activity is mediated by CREB3L1. To do this, *Creb3l1*-knockdown AtT20 cell lines were established by transduction of lentivirus containing *Creb3l1* shRNA. Control cells expressed a non-targeting shRNA. The knockdown cells were selected using puromycin treatment. Successful knockdown was demonstrated at the *Creb3l1* mRNA level by qPCR, and at the protein level by CREB3L1 immunoblotting (Fig. 3c). We then examined the effect of FSK treatments on *Creb3l1* mRNA expression in the *Creb3l1* knockdown cell line (Fig. 3d). The results showed that, from its diminished baseline, *Creb3l1* mRNA increased in abundance in response of FSK, suggesting that the knockdown cell line is still able to respond to FSK, but the level of *Creb3l1* mRNA expression is massively reduced compared to the control shRNA cell line. We then transfected the *Creb3l1* knockdown AtT20 cells with the luciferase reporter gene plasmids containing 1 kb of the *Avp* promoter, and asked about the response to FSK (Fig. 3e). Whilst FSK treatment robustly increased luciferase activity driven by the 1 kb *Avp* promoters in the control non-targeting shRNA cell line, knockdown of *Creb3l1* substantially diminished this effect, strongly suggesting that the effect of FSK-induced activation of the *Avp* promoter is mediated by *Creb3l1*.

### *Creb3l1* expression in the male rat PVN and SON in response to acute hyperosmotic stress

**Fig. 4:**
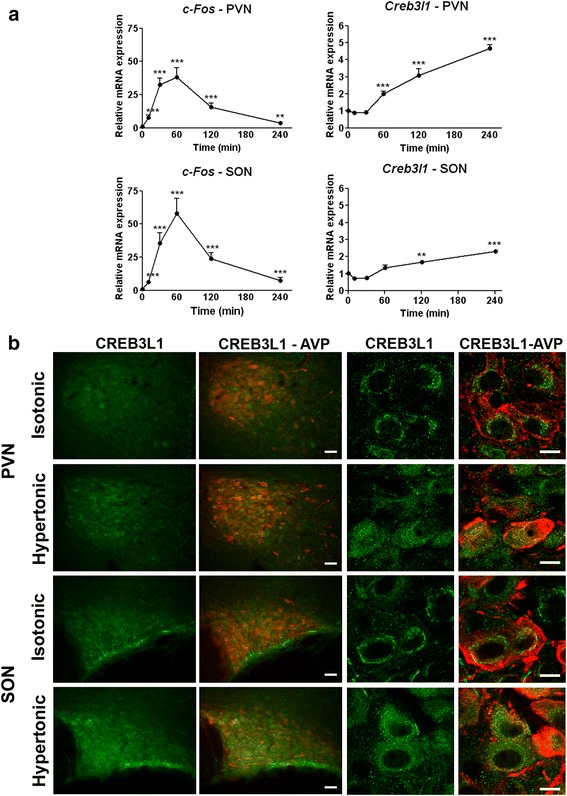
*Creb3l1* expression and localisation in rat PVN and SON in response to acute hyperosmotic stress. Rats were i.p. injected with hypertonic solution (1.5 M NaCl 1.5 ml/100 g body weight). **a** The level of *c-Fos* and *Creb3l1* gene expression was examined by qPCR in rat PVN and SON (*n* = 5). **b** Immunofluorescent staining of CREB3L1 (green) and AVP (red) showed early activation of CREB3L1 protein in response of hypertonic injection. The high magnification images by confocal microscope show the change in localization of CREB3L1 from perinuclear area to cytoplasm and nucleus in hyperosmotic stress. Scale bar, 70 μm and 10 μm (for confocal). Error bar + SEM; **, *p* < 0.01; ***, *p* < 0.001 (One way ANOVA); PVN, paraventricular nucleus; SON, supraoptic nucleus; AVP, arginine vasopressin

*In vitro* study showed that *Creb3l1* expression significantly increased at 1 h after FSK treatment (Fig.1a). To examine how quick the activation of *Creb3l1* expression occur in the rat PVN and SON, acute hyperosmotic stress was induced by intraperitoneal (i.p) injection of hypertonic saline (1.5 M, 1.5 ml/100 g of body weight). Hyperosmotic stimulation has previously shown to increase cAMP level in the SON [8]. The expression of *c-Fos* mRNA, a marker for neuronal activity which is also a cAMP-responsive gene, was investigated by qPCR to demonstrate activation of the PVN and SON in response to this stimuli (Fig. 4a). The abundance of the *c-Fos* mRNA increased very rapidly (10 min) after injection, and levels progressively increased until peaking at 60 min. This was followed by a decline in both PVN and SON. A higher degree of increase of *c-Fos* was observed in SON compared to PVN. At 60 min, the point at which *c-Fos* mRNA levels peaked, a statistically significant increase in *Creb3l1* mRNA levels was observed. However, in contrast with *c-Fos*, levels did not thereafter decline; rather, the abundance of *Creb3l1* mRNA progressively increased until the end of the experiment (4 h). A higher level of *Creb3l1* mRNA expression was observed in the PVN compared to the SON (Fig. 4a). Immunostaining of CREB3L1 was performed in rat PVN and SON following hyperosmotic stress to investigate CREB3L1 expression and localization (Fig. 4b). Compared to the controls injected with isotonic saline, rats injected i.p. with hypertonic saline showed a higher level of CREB3L1-like material in both PVN and SON (4 h after injection). The high magnification confocal microscope images showed increased CREB3L1-like material, together with a change in localisation from the perinuclear area in isotonic controls, to cytoplasmic and nuclear staining in hypertonic injection, as previously observed following chronic osmotic stimulation [20].

### Glucocorticoids inhibit *Creb3l1* and *Avp* expression in rat hypothalamus

**Fig. 5:**
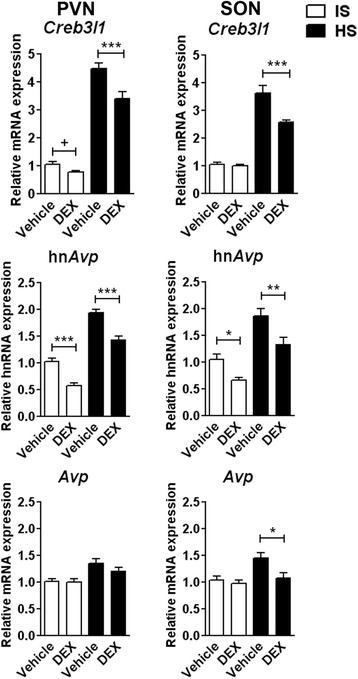
Effect of dexamethasone on *Creb3l1* expression in the rat PVN and SON. Rats were injected (i.p.) with either DEX or vehicle. Two hours after injection, isotonic (IS; 0.15 M NaCl) or hypertonic saline (HS; 1.5 M NaCl; 1.5 ml/100 g body weight) were administrated (i.p.). The brains were collected at 4 h after IS/HS injection. qPCR was performed to examine mRNA expression of *Creb3l1* and *Avp* (*n* = 6-8). Error bar + SEM; *, *p* < 0.05; **, *p* < 0.01; ***, *p* < 0.001 (Two-way ANOVA), +, *p* < 0.05 (independent sample unpaired Student’s *t*-tests). DEX, dexamethasone; PVN, paraventricular nucleus; SON, supraoptic nucleus; hn, heteronuclear; *Avp*, arginine vasopressin

To investigate glucocorticoid actions on *Creb3l1* and *Avp* gene expression *in vivo*, rats were injected with DEX before injection of isotonic or hypertonic saline (Fig. 5). At 4 h after saline injection, mRNA expression levels were examined in the PVN and SON. As expected, *Creb3l1* mRNA increased in response of hypertonic saline in both PVN and SON. DEX injection reduced hypertonic-induced *Creb3l1* mRNA expression in both PVN and SON compared to vehicle controls. Both basal and hypertonic-stimulated hn*Avp* expression was diminished by pretreatment with DEX in PVN and SON. DEX also decreased hypertonic stimulation of *Avp* mRNA in PVN and SON.

## Discussion

We recently identified CREB3L1 as a transcription factor capable of regulating *Avp* gene expression in the rat hypothalamus in response to the chronic hyperosmotic stressors of dehydration (1 and 3 days) and salt loading (1 and 7 days) [20]. However the connection between this newly identified transcription factor and the previously proposed mechanism of AVP transcription was unknown. Here we show that cAMP and glucocorticoids, regulators of *Avp* expression, similarly regulate *Creb3l1* expression. Importantly, we show that CREB3L1 mediates cAMP-induced *Avp* expression, which is subject to glucocorticoid inhibition. Therefore this study provides the link between CREB3L1 and previously proposed mechanism of AVP transcription.

An increase in cAMP has previously been reported in response of hyperosmotic stress in the PVN and SON [8]. There is some evidence showing that *Avp* expression is activated through cAMP pathways [6, 10, 25, 26]. To investigate the control of *Creb3l1* expression, and its relationship to *Avp* transcriptional control, AtT20 cells, which endogenously express *Creb3l1,* were used as a model. The expression of *Creb3l1* in these cells responded robustly to activation of cAMP by FSK treatment, with mRNA expression increasing after 1 h, similar to that observed following i.p. hypertonic saline administration in the rat. CREB3L1 protein levels also increased following FSK treatment, in parallel with the mRNA encoding it.

To confirm an effect of cAMP on *Creb3l1* expression in the hypothalamus, we used hypothalamic organotypic cultures to study *Creb3l1* expression in response to activation of cAMP pathways. Increase of *Avp* mRNA in response to FSK treatment was previously shown in this system [25]. The robust increase in *Creb3l1* expression induced by FSK confirmed responsiveness to cAMP pathways. The increase in hn*Avp* but not *Avp* mRNA after 4 h FSK treatment is consistent with many previous reports of *Avp* hnRNA being a sensitive measure of transcriptional activity for this gene [27, 28], without necessarily affecting the large pool of steady-state mature *Avp* mRNA. No activation of *c-Fos* expression at 24 h when *Avp* expression is still elevated is consistent with the previously proposed hypothesis by Arima and colleagues that *c-Fos* may be involved but is not essential for the induction of *Avp* transcription [29].

cAMP activation of AVP transcription is believed to be mediated by phosphorylation of CREB by PKA [6, 7]. However, a role for phosphorylated CREB in the control of *Avp* expression in an *in vivo* context has not been substantiated [11]. The shRNA-mediated *Creb3l1* knockdown reduced promoter activity, suggesting that cAMP-induced *Avp* transcription is in fact mediated by *Creb3l1*. To our knowledge no evidence of a relation between CREB3L1 and phosphorylated CREB has been reported; however a study in a trophoblast cell line proposed that cAMP-induced activation of *Creb3l1* is mediated by p38MAP kinase pathway [22]. The later induction of *Creb3l1* mRNA expression relative to the cAMP inducible immediate early gene *c-Fos*, both *in vitro* and *in vivo*, certainly suggests the involvement of an intermediary factor, potentially a transcription factor, though this remains to be determined. Furthermore, the previous studies on CREB3L1 also reported that CREB3L1 is activated by ER stress in several models [30–33]. Although we previously reported function of CREB3L1 in regulation of ER stress response genes in rat hypothalamus during hyperosmotic challenge [34], activation of CREB3L1 expression itself by ER stress has never been reported in the hypothalamic neurons.

To examine the dynamic changes in gene expression that govern the hypothalamic response to a hyperosmotic cue, we exploited the acute stimulus of i.p. administration of a hypertonic saline solution. As expected, expression of *c-Fos*, a cAMP-responsive immediate early gene, rapidly increased in both PVN and SON, peaking at 1 h and declining thereafter toward basal levels. The activation of *Creb3l1* transcription was slower than that of *c-Fos*, but unlike *c-Fos*, continued to increase from 1 h onwards. We, and others, have reported that hn*Avp* expression increases as early as 10 min and reaches a peak 30 min after hypertonic saline administration [35, 36]. This means that the increase in hn*Avp* transcription precedes the activation of *Creb3l1* mRNA expression in the PVN and the SON, as observed in the present study. Such a rapid increase in hn*Avp* expression implies that new protein synthesis may not be necessary to achieve this early response. We previously reported that CREB3L1 protein expression was most abundant in the Golgi of MCNs in euhydrated rats [20]. When it is activated, CREB3L1 is rapidly cleaved by regulated intramembrane proteolysis. The released N-terminal domain of the protein then enters the nucleus to regulate transcription of target genes [21]. One may speculate that the localisation of CREB3L1 in the Golgi of euhydrated rats supports a role for pre-existing CREB3L1 protein in the early activation of hn*Avp* transcription. A change in cellular and subcellular localisation of CREB3L1 was indeed evident 4 h after initiation of hyperosmotic stress (Fig. 4b), similar to our previous findings at the longer time point of 3 day dehydration [20]. Moreover, previous study in C6 glioma cells showed that ER stress inducer dithiothreitol can activate CREB3L1 at the protein level, while the mRNA level is unchanged. An activation signal was demonstrated to increase CREB3L1 stability by inhibiting degradation by the proteasome system [37]. Such a mechanism may facilitate the early function of CREB3L1 in the regulation of *Avp* transcription. Further investigation is needed to address this issue in the PVN and SON.

Inhibition of *Avp* expression by glucocorticoids has been described for many years, although the molecular mechanisms have remained unclear. Putative glucocorticoid responsive element (GRE) sites were identified in the rat and bovine *Avp* promoters (−622/-608 and within −300 to −155, respectively) [38, 39], but were not tested functionally. However, the promoters of many genes that are negatively regulated by glucocorticoids do not contain GRE sites, suggesting that the regulatory mechanisms of glucocorticoids are not only mediated by direct binding of glucocorticoid receptor to a GRE. The interaction of glucocorticoid receptor with transcription factors or co-factors is also believed to be a regulatory mechanism of glucocorticoids [40]. Here we show that DEX treatment inhibits both basal and FSK-stimulated *Creb3l1* expression in AtT20 cells. In organotypic cultures, DEX treatment inhibited FSK-induced *Avp* expression, in line with previous reports [24], and also FSK-induced *Creb3l1* expression, similar to our findings in AtT20 cells.

We were surprised that DEX treatment increased Avp promoter activity in AtT20 cells, disagreeing with previous studies in hypothalamic neuronal cells and human choriocarcinoma cell line JEG-3 that showed DEX treatment inhibits *Avp *promoter activity [10, 41]. AtT20 cells express glucocorticoid receptors [42] and activation of these receptors can positively and negatively regulate gene expression via the direct binding onto DNA or through direct protein-protein interaction [43]. One possibility is that DEX may alter other genes that influence *Avp* promoter activity in AtT20 cells that do not express *Avp*. This represents a limitation of performing promoter studies in AtT20 cells.

We next tested if peripheral administration of DEX in the animal could inhibit *Avp* and/or *Creb3l1* stimulation by hypertonic saline. Indeed, a single injection of DEX significantly diminished hypertonic-induced increases in hn*Avp* and *Creb3l1* expression in both PVN and SON. In agreement with DEX inhibition of *Avp* in PVN, withdrawal of glucocorticoids in the rat by adrenalectomy results in increased expression of *Avp* in PCNs, but not MCNs, and this effect can be reversed by administration of DEX or corticosterone [15, 17]. It is interesting that we observed a clear DEX inhibition of *Avp* expression in the whole PVN. Studies of glucocorticoid regulation in the PVN have largely opted for *in situ* hybridization rather than qPCR to measure *Avp* expression specifically in PCNs [15, 17, 44]. This approach prevents the large pool of *Avp* in MCNs masking the changes in gene expression in the smaller population of PCNs. However, our finding of DEX inhibition of *Avp* expression in both PVN and SON support the concept of DEX modulation of *Avp* expression may not only be evident in PCNs, but also in MCNs consistent with proposed functions of glucocorticoids in the control of water homeostasis [45]. It is known that modulation of excitatory and inhibitory synaptic transmission by glucocorticoids in the mouse PVN and SON is lost with conditional deletion of the glucocorticoid receptor [46]. However, the role of the glucocorticoid receptor on this DEX-induced regulation of *Creb3l1* expression in MCNs is not known. Therefore, our finding in AtT20 cells, organotypic cultures, and in vivo study suggest that DEX modulation of *Avp* expression is perhaps mediated through DEX-regulation of *Creb3l1* expression.

## Conclusion

In the long history of the characterisation of the regulatory mechanisms controlling expression of *Avp*, it has become accepted that the cAMP/PKA pathway is a major positive regulator for *Avp* transcription, whilst glucocorticoids have an inhibitory effect, but the molecular pathways mediating these effects have remained largely elusive [40]. Here, we provide evidence that *Creb3l1* is regulated by cAMP and glucocorticoids, and that this transcription factor may mediate the effects of these signaling pathways on *Avp* expression.

## Methods

### Animals

Male Sprague–Dawley rats weighing 250–300 g were used in this study. Rats were housed at a constant temperature of 22 °C and a relative humidity of 50–60 % (v/v) under a 14:10 h light/dark cycle. The rats were given free access to food and tap water, unless stated. Animal experiments were performed between 9 am—2 pm. All experiments were performed under a Home Office UK licence held under, and in strict accordance with, the provision of the UK Animals (Scientific Procedures) Act (1986); they had also been approved by the University of Bristol Animal Welfare and Ethical Review Board.

### Hypertonic stress experiment

To induce acute hyperosmotic stress, a single i.p. injection of 1.5 ml/100 g body weight of 1.5 M NaCl solution was performed. For gene expression study, rats were randomly allocated into 1 of 6 groups, control, 10 min, 30 min, 1, 2, and 4 hours (h) after administration of hypertonic saline. To investigate actions of glucocorticoid on gene expression in the hypothalamus, rats were injected i.p. with 0.5 ml 0.15 M NaCl (vehicle) or 0.5 ml of 1 mg/kg body weight dexamethasone (DEX; Sigma D2915) 2 h before Isotonic (0.15 M NaCl solution; 1.5 ml/100 g body weight) or hypertonic saline injection. After injection of hypertonic or isotonic saline rats were placed back in their home cages for 4 h, and water, but not food, was removed for the duration of the experiment. For RNA analyses, rats were killed by striking of the cranium, followed by decapitation, using a guillotine (Harvard Apparatus). Brains were removed and frozen on dry ice before being stored at −80 °C. For immunofluorescent study, rats were i.p. injected with hypertonic saline for 4 h. Isotonic saline solution was injected for the control group.

### Organotypic culture of rat hypothalamus

Organotypic cultures were performed following the protocol previously described [47]. Sprague–Dawley pups (P5-P7) were purchased from Harlan Laboratories (UK). Pups were decapitated using scissors, and brains were removed and incubated in cold-Hank’s solution for 5 min, and then dissected using the optic chiasm as a landmark to produce a hypothalamic block. The block was placed onto a Mcllwain Tissue Chopper and 400 μm sections containing the PVN were cut then transferred to ice-cold Hank’s solution and incubated for 1 h before being placed onto a Millicell Cell Culture Insert (30 mm, hydrophilic PTFE, 0.4 μm; Millipore PICM03050) in a 6-well tissue culture plate with 1.1 ml of culture medium. The culture sections were incubated at 37 °C, 5 % (v/v) CO_2_ for 14 days before performing an experiment. The culture medium was replaced with fresh media every 2 days, and 4 days before the experiment the medium was replaced with serum-free medium. For treatments, culture medium was replaced with serum-free medium containing 10 μM FSK (Sigma) and/or 100nM DEX. At appropriate time points, the membrane was frozen on dry ice, and then the PVN was punched out using a 1 mm diameter micropunch (Fine Scientific Tools). The punched tissue was dispersed into a 1.5 ml tube for RNA extraction using Qiazol Reagent with the Qiagen RNeasy kit as described below.

### Cells and treatments

AtT20/D16v-F2 cells (Sigma; 94050406) were cultured in DMEM (Sigma; D6546) supplemented with 10 % (v/v) heat-inactivated fetal bovine serum (Gibco), 2 mM L-glutamine and 100 unit/ml of penicillin-streptomycin. Cells were incubated at 37 °C in a humidified incubator with 5 % (v/v) CO_2_. For chemical treatments, cells were seeded onto tissue culture plates to 60–70 % confluence. After 18-24 h, the culture medium was replaced with medium containing 10 μM FSK (Sigma) and/or 100nM DEX.

To produce a *Creb3l1* knockdown cell line, AtT20 cells were transduced with a lentivirus containing shRNA targeting mouse *Creb3l1*. The *Creb3l1* shRNA sequence (CGGCTCAATGACTGTGAAAGA) was obtained from the RNAi consortium shRNA library. Sense and antisense oligonucleotides for shRNAs were synthesised (European MWG Operon) and cloned into lentiviral transfer vector pLKO.1 puro according to manufacturer’s guidelines (pLKO.1 puro was a gift from Bob Weinberg, Addgene plasmid 8453). A non-targeting shRNA sequence (ATCATGTTAGGCGTACGGACT) was used as a control. Virus particles were produced as previously described [20]. Twenty four hours after transduction, puromycin (2 μg/ml, Life technologies) was added to cell culture medium. The cells were cultured in presence of puromycin for two weeks before use in experiments. The level of knockdown was confirmed by quantitative PCR (qPCR) and immunoblotting of CREB3L1 (see below).

### RNA extraction and cDNA synthesis

Frozen brains were sliced into 60 μm coronal sections in a cryostat. Sections were mounted on glass slides and stained with 0.1 % (w/v) toludine blue/70 % ethanol then visualised on a light microscope until brain nuclei were visible, then PVN and SON samples were collected using a 1 mm micropunch. The optic chiasm (SON), or neurons staining at the PVN location lateral to the third ventricle (PVN), were used as a reference. PVN and SON samples were then dispensed into 1.5 ml tubes and kept on dry ice within the cryostat. Total RNA was extracted from punched samples by combining Qiazol Reagent with Qiagens RNeasy kit protocols (Qiagen). The punched samples were removed from dry ice and rapidly resuspended, by vortexing, in 1 ml Qiazol reagent. Following Qiazol phase separation with chloroform, 350 μl of the upper aqueous phase was removed, mixed with 350 μl 70 % (v/v) ethanol and applied to RNeasy columns. The remaining steps were performed as recommended by the manufacturer. For cDNA synthesis, 200 ng of total RNA was reverse transcribed using the Quantitect reverse transcription kit (Qiagen).

For *in vitro* studies, the culture medium was removed and the cells were lysed in 350 μl Qiazol Reagent (Qiagen). The lysate was mixed with 350 μl absolute ethanol and added directly into the Direct-zol™ RNA MiniPrep columns (Zymo research; R2052) and extraction continued following the manufacturer’s protocol. For cDNA synthesis, 500 ng of total RNA was reverse transcribed using the Quantitect reverse transcription kit (Qiagen).

### Real-time quantitative PCR analysis

Primers for rat *Creb3l1* (5’-GAGACCTGGCCAGAGGATAC-3’ and 5’-GTCAGTGAGCAAGAGAACGC-3’), rat *Avp* mRNA (5’-TGCCTGCTACTTCCAGAACTGC-3’ and 5’-AGGGGAGACACTGTCTCAGCTC-3’), rat *Avp* heteronuclear RNA (hn*Avp*) (5’-GAGGCAAGAGGGCCACATC-3’ and 5’-CTCTCCTAGCCCATGACCCTT-3’), rat *c-Fos* (5’-AGCATGGGCTCCCCTGTCA-3’ and 5’-GAGACCAGAGTGGGCTGCA-3’), rat *Rpl19* (5’-GCGTCTGCAGCCATGAGTA and 5’-TGGCATTGGCGATTTCGTTG-3’), mouse *c-Fos* (5’-TCCCCAAACTTCGACCATGA-3’ and 5’-GGCTGGGGAATGGTAGTAGG-3’), mouse *Creb3l1* (5’-ACAAACTGCAGGGGACATCA-3’ and 5’-GAGCTTGGTGGGGATAGGG-3’) and mouse *Gapdh* (5’-CAACTCCCACTCTTCCACCT-3’ and 5’-CTTGCTCAGTGTCCTTGCTG-3’) were synthesised by European MWG Operon. The optimisation and validation of primers was performed using standard ABI protocols. The cDNA from reverse transcriptase reaction was used as a template for subsequent PCRs, which were carried out in duplicate. Quantitative PCR was conducted in 12.5 μl reaction volumes using SYBR green master mix buffer (Roche) using an ABI StepOnePlus real time PCR system. For relative quantification of gene expression the 2^-ΔΔCT^ method was employed [48]. The internal control gene used for these analyses were the housekeeping genes *Rpl19* and *Gapdh*.

### Immunofluorescence

Rats were deeply anesthetised with sodium pentobarbitone (100 mg/kg i.p.) and transcardially perfused with 0.1 M phosphate buffered saline (PBS, pH 7.4) followed by 4 % (w/v) paraformaldehyde (PFA) in PBS. Brains were removed and post-fixed overnight in 4 % (w/v) PFA followed by 30 % (w/v) sucrose prepared in PBS. Coronal sections (40 μm) of the forebrain were cut on a cryostat and washed in PBS three times. Sections then were blocked in 5 % (v/v) horse serum prepared in PBS with 0.25 % (v/v) TritonX-100 (PBST) for 30 min and then incubated with goat anti-N-terminal CREB3L1 antibody (1:250; R&D systems, AF4080) and mouse monoclonal antibody recognising AVP neurophysin-II (NP-II, PS41; 1:200) [49] prepared in 1 % horse serum/PBST at 4 °C for 48 h. The sections were washed three times in PBS for 5 min and incubated with 1:500 dilution of anti-goat IgG-biotinylated secondary antibody in 1 % horse serum/PBST for 1 h at room temperature. Sections were washed three times for 5 min with PBS and incubated for 1 h with secondary antibodies conjugated with fluorophore (1:500 in 1 % horse serum/PBST; Alexa Fluor 488 streptavidin-conjugated and Alexa Fluor 594 donkey anti-mouse IgG (Invitrogen)). After three washes with PBS, sections were mounted onto glass slides with 0.5 % (w/v) gelatin, and sealed with VectorShields hard mounting media (Vector Laboratories Ltd.). For organotypic culture, the tissues on Millicell Cell Culture Insert were fixed with 4 % (w/v) PFA in PBS for 20 min and washed three times with PBS for 5 min. Tissues then were blocked and incubated with antibodies as described above. Images were captured on fluorescent microscope and confocal microscope (Leica). The specificity of anti-N-terminal CREB3L1 antibody in immunofluorescent staining was previously validated [20].

### Protein extraction and immunoblotting

Cells were washed twice with cold PBS pH 7.4 (Gibco; 10010–015), and harvested by scraping into RIPA buffer (1 % (w/v) IGEPAL CA-630 (Sigma I3021), 0.5 % (w/v) sodium deoxycholate, and 0.1 % (w/v) sodium dodecyl sulfate (SDS) prepared in PBS) supplemented with 1 mM PMSF and Protease Inhibitor Cocktail (Sigma; P8340). The lysate was incubated on ice for 15 min with vortexing every 5 min, followed by centrifugation at 10,000 × g for 10 min. Supernatants were collected and kept at −80 °C. For cytosolic and nuclear protein extraction, cells were harvested in 500 μl hypotonic buffer solution (for 5 × 10^6^ cells ; 20 mM Tris–HCl, pH 7.4, 10 mM NaCl, 3 mM MgCl_2_) supplemented with 1 mM PMSF and Protease Inhibitor Cocktail, then moved to 1.5 ml tube and gently resuspended by pipetting before being incubated on ice for 15 min. 25 μl of 10 % IGEPAL CA-630 was added to the lysate and vortexed for 10 seconds. The lysate was centrifuged at 1,000 × g at 4 °C for 10 min, and the supernatant was collected as the cytosolic protein. The pellet was resuspended in 50 μl of Cell Extraction Buffer (100 mM Tris, pH 7.4, 2 mM Na_3_VO_4_, 100 mM NaCl, 1 % (v/v) Triton X-100, 1 mM EDTA, 10 % (v/v) glycerol, 1 mM EGTA, 0.1 % (w/v) SDS, 1 mM NaF, 0.5 % (w/v) deoxycholate, 20 mM Na_4_P_2_O_7_, supplemented with 1 mM PMSF and Protease Inhibitor Cocktail) and incubated on ice for 30 min with vortexing at 10 min intervals. The lysate was centrifuged at 14,000 × g at 4 °C for 30 min, and the supernatant was collected as the nuclear fraction. Protein concentrations were determined using the Bradford assay (Bio-Rad).

For immunoblotting, proteins were separated by SDS-PAGE and transferred to 0.45 μm PVDF membranes (Millipore). The membranes were blocked with 5 % (w/v) skimmed-milk prepared in Tris-buffered saline-0.05 % Tween 20 (TBS-T) for 1 h at room temperature, followed by incubation with primary antibody diluted in 3 % (w/v) skimmed-milk in TBS-T at 4 °C overnight. This was followed by incubation with appropriate secondary antibody conjugated with horseradish peroxidase (HRP) at room temperature for 1 h. Signal was visualised using high sensitivity WESTAR EtaC or WESTAR SuperNova extreme sensitivity HRP Detection Substrate (Cyanagen). Primary antibodies used: a goat polyclonal anti-N-terminal CREB3L1 (1:1,000; R&D systems, AF4080) and a mouse polyclonal anti-GAPDH (1:20,000; Santa Cruz, sc-32233). Secondary antibodies used: rabbit anti-Goat IgG (whole molecule)–peroxidase antibody (Sigma: A5420) and rabbit anti-Mouse IgG (whole molecule)–Peroxidase antibody (Sigma: A9044). The specificity of anti-N-terminal CREB3L1 antibody in immunoblot assay was previously validated [20].

### Luciferase assays

For luciferase assays, we used 1 kb rat *Avp* promoter luciferase reporter construct cloned into pGL3 vector (Promega) as described previously [20]. AtT20 cells were seeded onto 12-well tissue culture plates. Next day, plasmids (1 μg pGL3-Avp promoter and 0.1 μg pRL-TK vector/well) were transfected into cells using lipofectamine LTX with Plus transfection reagent (Life Technologies) following the manufacturer’s protocol. For chemical treatments, the culture medium was replaced with fresh medium containing 10 μM FSK or DMSO (vehicle) 24 h after transfection. For DEX treatment, the cells were pre-treated with DMSO or 100nM DEX for 2 h followed by treatment with 10 μM FSK or DMSO for 4 h. Cells were harvested at appropriate time points. Luciferase assays were performed using the Promega Dual-Luciferase® Reporter Assay. Luciferase activity was measured in triplicate using a Lumat LB 9507 Luminometer (Berthold Technologies).

### Statistical analysis

All data are expressed as the mean + SEM. Statistical differences between experimental groups were evaluated using independent sample unpaired Student’s *t*-tests or One-way ANOVA with Tukey post test (for comparison of more than 2 groups). Two-way ANOVA with Bonferonni post hoc test was used to determine interactions between two independent variables on the dependent variable. If Bartlett’s test for equal variances has *p* < 0.01 in One-way ANOVA, data was log transformed before analysis. *p* < 0.05 was considered significant.

## Abbreviations

AVP: Arginine vasopressin
CREB: cAMP responsive element-binding protein
DEX: Dexamethasone
FSK: Forskolin
hnRNA: heteronuclear RNA
MCN: Magnocellular neuron
NP-II: Neurophysin-II
PVN: Paraventricular nucleus
RIP: Regulated intra-membrane proteolysis
SCN: Suprachiasmatic nucleus
shRNA: small hairpin RNA
SON: Supraoptic nucleus
